# The prevalence of cardiometabolic multimorbidity and its associations with health outcomes among women in China

**DOI:** 10.3389/fcvm.2023.922932

**Published:** 2023-02-09

**Authors:** Yang Zhao, Huan Zhang, Xiaoyun Liu, Allissa Desloge, Qian Wang, Siqi Zhao, Lili Song, Ioanna Tzoulaki

**Affiliations:** ^1^The George Institute for Global Health, University of New South Wales, Sydney, NSW, Australia; ^2^The George Institute for Global Health, Beijing, China; ^3^Department of Infectious Disease Epidemiology, London School of Hygiene and Tropical Medicine, London, United Kingdom; ^4^China Centre for Health Development Studies, Peking University, Beijing, China; ^5^School of Public Health, University of Illinois Chicago, Chicago, IL, United States; ^6^Yeda Hospital of Yantai, Yantai, Shandong, China; ^7^Yantaishan Hospital of Yantai, Yantai, Shandong, China; ^8^Yantai Sino-French Friendship Hospital, Yantai, Shandong, China; ^9^Department of Epidemiology and Biostatistics, Imperial College London, London, United Kingdom; ^10^Department of Hygiene and Epidemiology, University of Ioannina School of Medicine, Ioannina, Greece

**Keywords:** cardiometabolic disease, multimorbidity, health outcomes, women, Chinese adults

## Abstract

**Objective:**

In China, a limited number of studies focus on women and examine the effect of cardiometabolic multimorbidity (defined as the presence of two or more cardiometabolic diseases) on health outcomes. This research aims to investigate the epidemiology of cardiometabolic multimorbidity and the association of cardiometabolic multimorbidity with long-term mortality.

**Methods:**

This study used data from the China Health and Retirement Longitudinal Study between 2011 and 2018, which includes 4,832 women aged 45 years and older in China. Poisson-distributed Generalized Linear Models (GLM) were applied to examine the association of cardiometabolic multimorbidity with all-cause mortality.

**Results:**

Overall, the prevalence of cardiometabolic multimorbidity was 33.1% among the total sample of 4,832 Chinese women, and increased with age, ranging from 28.5% (22.1%) for those aged 45–54 years to 65.3% (38.2%) for those aged ≥75 years in urban (rural) areas. Compared with the group of none and single disease, cardiometabolic multimorbidity was positively associated with all-cause death (RR = 1.509, 95% CI = 1.130, 2.017), after adjusting socio-demographic and lifestyle behavioral covariates. Stratified analyses revealed that the association between cardiometabolic multimorbidity and all-cause death was only statistically significant (RR = 1.473, 95% CI = 1.040, 2.087) in rural residents, but not significant in urban residents.

**Conclusion:**

Cardiometabolic multimorbidity is common among women in China and has been associated with excess mortality. Targeted strategies and people-centered integrated primary care models must be considered to more effectively manage the cardiometabolic multimorbidity shift from focusing on single-disease.

## 1. Introduction

Metabolic disease is associated with the high risk of cardiovascular diseases (1, 2) and all-cause mortality (3–5) has been a major global challenge. In 2019, one-third of all deaths worldwide were due to cardiovascular disease and the cardiovascular diseases, especially coronary heart disease and stroke, is the leading cause of death worldwide (3). The disease burden of cardiometabolic multimorbidity (defined as the coexistence of two or more cardiometabolic diseases) is rising rapidly. In China and low-and middle income countries (LMICs), the number of people experiencing cardiometabolic multimorbidity has increased rapidly over the past few decades (6–8). In European countries and the United States, multimorbidity is associated with higher healthcare service utilization, poorer health outcomes, and mortality, challenging the single-disease framework of most healthcare configurations (9, 10).

Although several studies have been conducted in high-income countries (HICs) on the burden and effect of multimorbidity, this topic remains an emerging research area of research in LMICs (11–13). Recently, only a few studies in certain regions of China have explored this topic (14–16). For example, the Chinese Electronic Health Records Research in Yinzhou (CHERRY) study focusing on Yinzhou County, which examined the burden of cardiometabolic multimorbidity and mortality risk among 1 million subjects in China (16).

Most of previous studies investigated the relationships between some single chronic conditions alone and health outcomes. There is limited evidence on the long-term changes of health related outcomes in individuals who are suffering from cardiometabolic multimorbidity (3–5). In China, there is no study focusing on females and estimated the impact of cardiometabolic multimorbidity on long-term mortality using nationally representative data (17–21). Therefore, the objective of this research is to investigate the prevalence of cardiometabolic multimorbidity among Chinese women and the association of cardiometabolic multimorbidity with all-cause mortality, using nationally representative population-based cohort data.

## 2. Materials and methods

### 2.1. Data source

A nationally representative population-based cohort study was designed, using longitudinal data from the baseline and newest wave of China Health and Retirement Longitudinal Study (CHARLS) conducted from 2011 to 2018. The aim of CHARLS is to collect a set of high-quality micro-data representing families and individuals of middle-aged and elderly people aged 45 and above in China. The CHARLS baseline survey was launched in 2011, covering 150 counties, 450 villages/communities as primary sampling units (PSUs), and over 17,000 individuals in about 10,000 households. These samples will then be tracked every 2 to 3 years. Data were collected in a survey in which four-stage stratified cluster sampling was used to select eligible individuals. Details of the procedures involved in CHARLS and its multistage stratified sampling are described elsewhere (22). The CHARLS questionnaire includes: basic socio-demographic information, family structure and income, health status, health service utilization and expenditure, retirement and pensions, anthropometric measurements and biomarkers. Written informed consent was obtained from all participants (22).

The baseline of CHARLS collected a total of 17,708 participants, including 9,230 adult women, with the respondent rate above 80%. The fourth wave of CHARLS survey successfully re-interviewed 7,593 females in 2018. This study identified 5,538 participants with biomarker information and blood test. After deleting the individuals aged below 45 years and those respondents with missing values for the outcome variable and covariates of interest, 4,832 individuals who performed the two wave surveys were included (The sample selection flowchart was shown in Supplementary Figure 1).

### 2.2. Measures

We counted the number of chronic diseases for each participant, identifying those with multimorbidity (23, 24). Cardiometabolic multimorbidity was defined as the presence of two or more cardiometabolic diseases included for each participant (17, 20, 21). A total of seven cardiovascular and metabolic diseases were used to measure multimorbidity. Hypertension, diabetes, dyslipidaemia, hyperuricemia and central obesity were measured by biomarkers or blood test information. Another two non-fatal cardiometabolic diseases, including heart disease and stroke, were ascertained *via* self-reports of a physician or health professional diagnosis of heart disease or stroke.

In this study, the hypertension was defined as systolic blood pressure ≥140 mmHg and/or diastolic blood pressure ≥90 mmHg, and/or taking antihypertensive drugs for elevated blood pressure (25). The diabetes was defined as (1) fasting blood glucose level ≥126 mg/dL (7.0 mmol/L); and/or (2) HbA1c concentration ≥6.5%; and/or (3) insulin therapy and/or medication for elevated blood glucose (26). The dyslipidemia was defined as (1) total cholesterol ≥240 mg/dL (6.22 mmol/L); and/or (2) low-density lipoprotein cholesterol ≥160 mg/dL (4.14 mmol/L); and/or (3) high-density lipoprotein cholesterol Protein cholesterol <40 mg/dL (1.04 mmol/L); and/or (4) triglycerides ≥200 mg/dL (2.26 mmol/L); and/or (5) taking anti-dyslipidemic drugs (27). The hyperuricemia is defined as a serum uric acid concentration greater than 7.0 mg/dL in men and greater than 6.0 mg/dL in women (28). The central obesity was defined as a waist circumference greater than 85 cm in female participants with a body mass index (BMI) ≥30 kg/m^2^ (29). The presence of heart disease and stroke, respectively, was determined by two questions in CHARLS survey: the participants were asked “Have you been diagnosed with heart attack, coronary heart disease, angina, congestive heart failure, or other heart problems by a doctor” and “Have you been diagnosed with stroke by a doctor?”

Regarding the health related outcome variable, all-cause mortality during the period of 2011–2018 was used as the primary outcome. The death information of participants at baseline was collected in sequential surveys and the last follow-up was conducted in 2018.

### 2.3. Statistical analysis

Poisson-distributed Generalized Linear Models (GLM) were conducted to determine the longitudinal association of Cardiometabolic multimorbidity with all-cause mortality. Models are adjusted on age, marital status, educational level, residence place, region, and social health insurance at baseline (model 1) and other confounding factors (smoking, drinking alcohol, other non-communicable diseases (NCDs), and depression) (model 2). Depressive disorder was measured by the 10-item Center for Epidemiologic Studies Depression Scale (CES-D 10), which has been identified as a valid and reliable useful mental health assessment tool in China (30).

We performed stratified analyses to further investigate urban-rural differences of the association of cardiometabolic multimorbidity with all-cause death. In terms of the multivariable regression analyses, relative risk ratios (RR) and 95% confidence intervals (CI) were reported. We performed the sensitivity analysis on relationships between the single components of cardiometabolic syndrome and all-cause mortality. We also conducted the regression analyses by using a newer cut-off of 5.1 mg/dl for the definition of hyperuricemia for females (31). All analyses were weighted to account for the multi-stage probability-proportionate-to-size sampling (PPS) design of CHARLS, and conducted using Stata Version 16.0 (Stata Corp., College Station, TX, USA). *P*-values < 0.05 were deemed statistically significant.

## 3. Results

Our analysis included 4,832 females from the CHARLS in China. The mean age of respondents was 57.7 years at the baseline survey. Among the participants, 60.1% of the respondents were illiterate, 65.1% of individuals living in rural areas and 93.6% of individuals with social medical insurances. Among Chinese women, the proportion of current smoking and alcohol drinking was 5.9 and 12.2%, respectively. The prevalence of depression disorder was 44.3% (Table 1).

**TABLE 1:** Characteristics of participants, the prevalence of single condition and CMD multimorbidity.

Variables	*N* (Percentage)^a^		Prevalence of single disease^b^ (95% CI)			Prevalence of CMD multimorbidity (95% CI)		
All	4,832	100.0	34.1%	32.2%	36.0%	33.7%	31.6%	35.9%
**Age, years**								
45–55	1,900	39.3	32.4%	29.2%	35.8%	25.1%	21.3%	29.4%
55–65	1,903	39.4	34.9%	32.3%	37.6%	35.7%	33.1%	38.4%
65–75	786	16.3	35.2%	30.8%	39.8%	44.9%	40.5%	49.5%
≥ 75	243	5.0	37.2%	29.0%	46.1%	50.4%	41.0%	59.7%
**Marital status**								
Married and partnered	4,200	86.9	33.6%	31.6%	35.7%	32.5%	30.2%	35.0%
Unmarried and others	632	13.1	36.6%	31.9%	41.6%	40.7%	35.9%	45.7%
**Education level**								
Illiterate	2,901	60.0	36.5%	34.3%	38.7%	33.8%	31.6%	36.0%
Primary school	858	17.8	31.2%	27.3%	35.3%	37.5%	32.9%	42.3%
Secondary school	726	15.0	31.0%	25.8%	36.7%	30.1%	25.0%	35.7%
College and above	347	7.2	31.7%	24.1%	40.4%	33.5%	22.6%	46.5%
**Residence place**								
Urban	1,685	34.9	30.7%	27.4%	34.3%	39.2%	35.0%	43.5%
Rural	3,147	65.1	36.8%	34.9%	38.7%	29.2%	27.5%	31.0%
**Region**								
East	1,780	36.8	33.9%	30.4%	37.5%	33.0%	28.9%	37.3%
Central	1,852	38.3	34.5%	31.7%	37.3%	35.8%	33.2%	38.5%
West	1,200	24.8	33.7%	30.4%	37.2%	32.0%	28.0%	36.2%
**Social health insurance**								
No	311	6.4	35.2%	28.6%	42.4%	31.9%	24.9%	39.9%
Yes	4,521	93.6	34.0%	32.0%	36.0%	33.9%	31.7%	36.2%
**Smoking**								
No	4,549	94.1	34.0%	32.1%	36.1%	33.5%	31.3%	35.8%
Yes	283	5.9	34.2%	27.6%	41.5%	38.1%	31.0%	45.8%
**Alcohol drinking**								
No	4,241	87.8	33.6%	31.6%	35.7%	34.9%	32.6%	37.4%
Yes	591	12.2	37.4%	32.5%	42.5%	24.8%	20.8%	29.3%
**Other NCDs**								
No	1,958	40.5	33.2%	29.9%	36.7%	31.3%	27.4%	35.5%
Yes	2,874	59.5	34.7%	32.5%	36.9%	35.5%	33.3%	37.8%
**Depression**								
No	2,690	55.7	33.7%	31.0%	36.5%	32.4%	29.2%	35.7%
Yes	2,142	44.3	34.6%	32.2%	37.1%	35.7%	33.2%	38.3%

a, *N* and percentages were based on study samples (unweighted); b, weighted prevalence of single disease and cardiometabolic multimorbidity. CMD, cardiometabolic disease.

**FIGURE 1:**
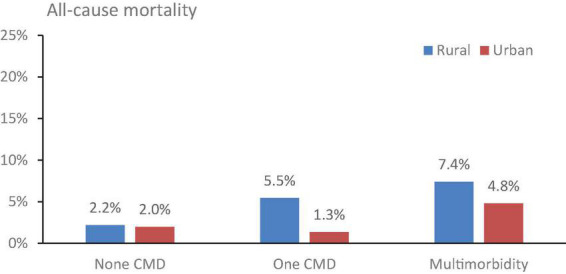
The all-cause mortality from 2011 to 2018 by the number of disease and residence place. CMD, cardiometabolic disease.

Table 2 showed the prevalence of main cardiometabolic diseases and multimorbidity across age group and residence place. Among the Chinese women, the prevalence of cardiometabolic multimorbidity was 33.1%, and raised with age, ranging from 28.5% (22.1%) for those aged 45–54 years to 65.3% (38.2%) for those aged 75 years and above in urban (rural) areas. Over 30% of Chinese women experiencing hypertension and hyperlipidemia. The prevalence of heart disease, diabetes, concentric obesity, hyperuricemia and stroke was 15.9, 14.7 5.5, 4.5, and 2.6%, respectively. The prevalence of hypertension, hyperlipidemia, diabetes as well as stroke increased with age among urban citizens and was commonly higher than those of rural residents. Figure 1 showed a higher proportions of all-cause death among individuals in rural areas compared to individuals in urban areas.

**TABLE 2:** The proportion of cardiometabolic diseases and multimorbidity among Chinese adults by age group and residence place (*N* = 4,832).

CMD and multimorbidity	Total		Rural				Urban			
	***n***	**%**	**45–55**	**55–65**	**65–75**	**≥ 75**	**45–55**	**55–65**	**65–75**	**≥ 75**
**Single disorder**										
Hypertension	1,641	34.0	22.0	33.2	49.5	59.7	24.0	37.5	49.5	61.2
Hyperlipidemia	1,986	41.1	34.9	40.5	40.9	42.4	40.1	51.2	51.9	57.7
Diabetes	708	14.7	11.2	15.3	14.0	16.7	12.4	19.3	21.3	26.3
Hyperuricemia	216	4.5	2.3	3.6	8.2	11.6	3.7	8.4	8.5	6.4
Concentric obesity	267	5.5	4.4	4.8	3.5	2.6	5.5	7.9	7.3	8.0
Heart disease	768	15.9	12.2	14.9	15.4	8.3	10.5	20.7	29.5	29.1
Stroke	127	2.	1.9	3.3	3.6	1.4	1.5	3.0	3.3	6.4
Overall multimorbidity	1,601	33.1	22.1	31.1	39.0	38.2	28.5	41.8	52.0	65.3

Values are unweighted counts and weighted percentages unless otherwise indicated. Overall multimorbidity refers to any two or more cardiometabolic diseases included in this study. CMD, cardiometabolic disease.

Table 3 revealed the relationship between cardiometabolic multimorbidity and health-related outcomes. Cardiometabolic multimorbidity was positively associated with all-cause death (RR = 1.509, 95% CI = 1.130, 2.017), after adjusting socio-demographic and lifestyle behavioral covariates. In women with ages above 65 years, depression and those without social health insurance were more likely to have the risk of death (Table 3). The stratified analysis showed that he association of cardiometabolic multimorbidity with all-cause death was only statistically significant (RR = 1.473, 95% CI = 1.040, 2.087) in rural residents, but not significant in urban residents (Table 4). The sensitivity analyses suggested consistent results (Supplementary Tables 1, 2). For the single disorders, stroke, heart disease and diabetes showed a statistically significant association with all-cause mortality, after adjusting socio-demographic and lifestyle behavioral covariates (Supplementary Table 3).

**TABLE 3:** Association of CMD multimorbidity with all-cause death.

Variable (reference)	Model 1					Model 2		
	**RR**	**95% CI**		***P-*value**	**RR**	**95% CI**		***P*-value**
CMD multimorbidity (single disorder)	1.529	1.146	2.039	0.004	1.509	1.130	2.017	0.005
**Age (45–59 years)**								
55–65	1.539	0.887	2.672	0.125	1.514	0.872	2.630	0.140
65–75	6.035	3.592	10.140	<0.001	5.970	3.552	10.036	<0.001
≥ 75	13.694	7.751	24.193	<0.001	13.362	7.566	23.599	<0.001
Marital status (married)	1.340	0.963	1.863	0.082	1.312	0.944	1.824	0.106
**Education level (Illiterate)**								
Primary school	0.837	0.536	1.307	0.435	0.851	0.544	1.330	0.479
Secondary school	1.167	0.677	2.013	0.578	1.209	0.701	2.086	0.496
College and above	0.297	0.072	1.228	0.094	0.317	0.077	1.315	0.114
Residence place (urban)	1.164	0.850	1.595	0.345	1.137	0.828	1.561	0.428
**Region (east)**								
Central	1.094	0.783	1.528	0.600	1.066	0.762	1.492	0.708
West	1.278	0.893	1.829	0.180	1.265	0.877	1.826	0.209
Social health insurance (no)	0.602	0.393	0.922	0.020	0.597	0.390	0.914	0.018
Smoking (no)	–	–	–	–	1.274	0.758	2.143	0.360
Alcohol drinking (no)	–	–	–	–	0.966	0.618	1.511	0.880
Other NCDs (no)	–	–	–	–	0.916	0.677	1.239	0.569
Depression (no)	–	–	–	–	1.406	1.044	1.894	0.025

Models are adjusted on age, marital status, educational level, residence place, region, and social health insurance at baseline (model 1) and other confounding factors (smoking, alcohol drinking, other NCDs, and depression) (model 2). CMD, cardiometabolic disease; RR, relative risk ratio; CI, confidence interval; Other NCDs, other non-communicable diseases excluding the seven cardiometabolic diseases in this study.

**TABLE 4:** Association of CMD multimorbidity with all-cause death among participants living the rural and urban area.

Variable (reference)	Rural					Urban		
	**RR**	**95% CI**		***P-*value**	**RR**	**95% CI**		***P-*value**
CMD Multimorbidity (single disorder)	1.473	1.040	2.087	0.029	1.571	0.920	2.681	0.098
**Age (45–59 years)**								
55–65	1.404	0.699	2.819	0.340	1.649	0.657	4.139	0.28
65–75	6.638	3.520	12.516	<0.001	4.606	1.833	11.571	0.001
≥ 75	14.157	7.077	28.322	<0.001	11.690	4.196	32.570	<0.001
Marital status (married)	1.331	0.893	1.982	0.160	1.257	0.694	2.277	0.450
**Education level (Illiterate)**								
Primary school	0.732	0.398	1.347	0.316	1.128	0.570	2.233	0.729
Secondary school	1.076	0.477	2.426	0.860	1.355	0.631	2.910	0.436
College and above	0.674	0.091	4.976	0.699	0.248	0.033	1.874	0.177
**Region (east)**								
Central	0.965	0.642	1.449	0.862	1.265	0.689	2.324	0.449
West	1.175	0.755	1.828	0.476	1.607	0.821	3.142	0.166
Social health insurance (no)	0.611	0.356	1.047	0.073	0.623	0.305	1.274	0.195
Smoking (no)	1.089	0.548	2.163	0.807	1.645	0.732	3.693	0.228
Alcohol drinking (no)	1.193	0.739	1.927	0.471	0.358	0.087	1.476	0.155
Other NCDs (no)	0.774	0.537	1.113	0.167	1.321	0.761	2.292	0.323
Depression (no)	1.290	0.901	1.847	0.165	1.711	1.009	2.902	0.046

Models are adjusted on age, marital status, educational level, region, social health insurance, smoking, alcohol drinking, other NCDs, and depression. CMD, cardiometabolic disease; RR, relative risk ratio; CI, confidence interval; Other NCDs, other non-communicable diseases excluding the seven cardiometabolic diseases in this study.

## 4. Discussion

This research analyzed nationally representative longitudinal data from the CHARLS to examine the prevalence of cardiometabolic multimorbidity and its association of cardiometabolic multimorbidity with all-cause death among middle-aged and elderly Chinese females. This study revealed that cardiometabolic multimorbidity is common among Chinese women, especially among the elderly and urban citizens. We found that cardiometabolic multimorbidity was associated with excess mortality. Furthermore, the association between cardiometabolic multimorbidity and all-cause death was only statistically significant in rural residents, but not significant in urban residents.

The cardiometabolic multimorbidity prevalence estimated by this study was 33.1% among middle-aged and older Chinese women in 2011. For general adults in China, there were studies showing a lower prevalence of metabolic disorder (16.5% in 2000 and 23.3% in 2009) (7). A meta-analysis in the basis of studies from mainland China also showed that the pooled prevalence of metabolic syndrome was 24.5% (32). Those evidence suggested that China is experiencing an emerging cardiometabolic disease epidemic, which may be related to the accelerated changes in dietary patterns and lifestyle behaviors due to demographic and socioeconomic transitions during the past decades (33). Moreover, there are many studies revealing a high prevalence of cardiometabolic syndromes in western developed countries and other regions in Asia, such as the United States (35%), Iran (37%), and Turkey (44%) (34–36). Variations in cardiometabolic syndrome prevalence between LMICs and HICs could be because of real differences across countries, and might also be due to potentially different definitions and sampling methods (36, 37).

The results from this research also suggested that cardiometabolic multimorbidity increased with the individual’s age and the people living in urban areas had a higher proportion of cardiometabolic multimorbidity than those rural persons in China. In line with previous findings, the burden of cardiometabolic disease increases with age due to the declining trends in cardiometabolic function (34, 38). Urban residents experiences a higher proportion of cardiometabolic multimorbidity than rural residents (32, 39), which could be attributed to physical inactivity, sedentary, unhealthy diet (such as excessive intake of high-calorie food, fat and salt) during the process of urbanization and economic development (39–41).

A few of studies have suggested that multiple chronic diseases have a significant impact on health related outcomes and mortality (10, 16–18). These findings are consistent with our previous studies in China (16, 19–21), United States (10, 42, 43), Japan (44), and Europe (45), where multimorbidity presented in health-related outcomes and deaths (10, 16). Cardiovascular and metabolic diseases have been revealed by several studies to play a dominant role in multiple multimorbidity patterns (41). The association between single cardiometabolic disease and negative health outcomes and mortality has also been documented (46). This research further provides new evidence that significant relationship between cardiometabolic multimorbidity and long-term mortality among adult women in China. We also performed the regression analyses by using a recently lower cut-off of 5.1 mg/dl for the definition of hyperuricemia for females (31), which also suggested consistent results.

Stratified analyses of this study revealed a significant association between cardiometabolic multimorbidity and all-cause death in rural residents, but there was not a significant association in urban residents while they had a high prevalence of cardiometabolic multimorbidity. Previous studies from China have documented disparities in the healthcare access and service utilization between residents living in rural and urban areas (47). Patients in rural areas have less geographic access to health-care than those patients living in urban areas. Rural areas in China could be more prone to healthcare provider shortages and lack of medical resources (48, 49). Rural residents usually face greater financial barriers and relatively low-quality health care (50). Thus, early screening of high-risk groups of cardiometabolic multimorbidity should be strengthened, and targeted preventive measures should be taken further. A strong people-centered primary care system needs to be oriented toward cardiometabolic diseases and multimorbidity, which has been shown to be the most cost-effective way to manage chronic diseases (51–54).

Establishing a multidisciplinary team composed of clinical physicals, geriatricians, pharmacists, nurses, nutritionists, rehabilitation physiotherapists, social workers, patients and their families is a potential model for dealing with multimorbidity issues, following the “people-centered principle” of managing multimorbidity. In the process of achieving the “Healthy China 2030” strategic goals (55) and Sustainable Development Goals, considering the increasing burden of multimorbidity populations and its long-term impact in future, it need more attention from clinicians, researchers and health policy makers from China as well as other countries with economies in transition.

To our best knowledge, this is the first nationally representative cohort study that focused on adult women and investigated associations of cardiometabolic multimorbidity with long-term mortality in China that examined the rural-urban differences in the relationships, by using longitudinal data with cardiometabolic biomarkers for the disease diagnosis. This study also has some limitations. First, while we identify several cardiometabolic diseases on the basis of biomarkers, the partial use of self-reported measures of heart disease and stroke could underestimate the prevalence of these chronic diseases. Second, we estimated the prevalence of cardiometabolic multimorbidity and its association with health outcomes by simply counting the number of chronic conditions, hence the accuracy of findings may be affected by the types of cardiometabolic multimorbidity. CHARLS only collected the data for calculating all-cause mortality and we could not further analyze the association between multimorbidity and mortality due to stroke or cardiovascular diseases. Third, there was only a 7-year follow-up period of CHARLS data and long-term impacts on health-related outcomes due to multimorbidity should be examined further. Finally, this study only analyzed the middle-aged and older Chinese women due to the CHARLS data collection, which may affect the generalizability of the findings from this study. The prevalence of cardiometabolic multimorbidity and its relationships with more health related outcomes among younger populations should be considered for future studies.

## 5. Conclusion

Cardiometabolic multimorbidity is common among women in China and has been associated with excess mortality. Health-care delivery models need to shift from focusing on single-disease to effectively managing cardiometabolic multimorbidity. Targeted strategies and people-centered integrated primary care models must be considered to more effectively manage the cardiometabolic multimorbidity shift from focusing on single-disease. Medical resources need to be prioritized to screen, prevent and treat cardiometabolic diseases as well as multimorbidity, especially in rural areas.

## Data availability statement

The original contributions presented in this study are included in this article/Supplementary material, further inquiries can be directed to the corresponding author.

## Ethics statement

The studies involving human participants were reviewed and approved by The Biomedical Ethics Review Committee of Peking University approved the CHARLS study (approval number: IRB00001052–11015), and all interviewees were required to provide informed consent. The patients/participants provided their written informed consent to participate in this study.

## Author contributions

YZ conceived and designed the study and did the initial analysis. LS supervised data analysis. YZ and SZ wrote the first draft of the manuscript. HZ, XL, AD, QW, LS, and IT critically revised the first draft. All authors reviewed, edited and commented on multiple versions of the manuscript.
