# Supplementary material for: The prevalence of cardiometabolic multimorbidity and its associations with health outcomes among women in China
Source: Front Cardiovasc Med. 2023 Feb 9;10:922932. doi: 10.3389/fcvm.2023.922932 (PMC9947472; doi:10.3389/fcvm.2023.922932)
Supplement: Supplementary file 1 [file Data_Sheet_1.docx]

**Appendix**

9230 female individuals recruited in 2011

2761 excluded*:

Missing marriage status (n=4)

Missing education (n=1)

Missing insurance (n=89)

Missing smoking (n=73)

Missing alcohol drinking (n=53)

Missing CES-D (n=984)

Missing blood test (n=2055)

8841 eligible individuals at age 45 years and above

4,832 included in analysis of death

389 excluded due to age below 45 years

7593 CHARLS participants followed up

331 CHARLS participants died

**Figure S1**. Flowchart of sample selection

*Some patients were missing more than one type of data. CES-D, Center for Epidemiologic Studies Depression Scale.

**Table S1**. Association of CMD multimorbidity with all-cause death

| **Variable (reference)** |  | **Model 1** | |  |  |  | **Model 2** | |  |
| --- | --- | --- | --- | --- | --- | --- | --- | --- | --- |
|  | RR | 95% CI | | P value |  | RR | 95% CI | | P value |
| **CMD Multimorbidity** (single disorder) | 1.465 | 1.111 | 1.930 | 0.007 |  | 1.485 | 1.109 | 1.987 | 0.008 |
| **Age** (45-59 years) |  |  |  |  |  |  |  |  |  |
| 55-65 | 1.447 | 0.859 | 2.438 | 0.165 |  | 1.509 | 0.869 | 2.621 | 0.144 |
| 65-75 | 5.502 | 3.367 | 8.990 | <0.001 |  | 5.954 | 3.540 | 10.014 | <0.001 |
| ≥75 | 13.125 | 7.677 | 22.439 | <0.001 |  | 13.182 | 7.455 | 23.310 | <0.001 |
| **Marital status** (married) | 1.356 | 0.989 | 1.860 | 0.059 |  | 1.322 | 0.952 | 1.837 | 0.096 |
| **Education level** (Illiterate) |  |  |  |  |  |  |  |  |  |
| Primary school | 0.778 | 0.500 | 1.211 | 0.266 |  | 0.854 | 0.546 | 1.335 | 0.488 |
| Secondary school | 1.156 | 0.690 | 1.938 | 0.582 |  | 1.212 | 0.703 | 2.092 | 0.489 |
| College and above | 0.266 | 0.064 | 1.098 | 0.067 |  | 0.318 | 0.077 | 1.316 | 0.114 |
| **Residence place** (urban) | 1.149 | 0.850 | 1.554 | 0.365 |  | 1.135 | 0.827 | 1.560 | 0.433 |
| **Region (**east**)** |  |  |  |  |  |  |  |  |  |
| Central | 1.187 | 0.866 | 1.627 | 0.286 |  | 1.071 | 0.766 | 1.499 | 0.687 |
| West | 1.213 | 0.854 | 1.723 | 0.281 |  | 1.262 | 0.875 | 1.822 | 0.213 |
| **Social health insurance** (no) | 0.665 | 0.438 | 1.009 | 0.055 |  | 0.602 | 0.393 | 0.923 | 0.020 |
| **Smoking** (no) | - | - | - | - |  | 1.272 | 0.756 | 2.138 | 0.364 |
| **Alcohol drinking** (no) | - | - | - | - |  | 0.957 | 0.612 | 1.496 | 0.848 |
| **Other NCDs** (no) | - | - | - | - |  | 0.915 | 0.677 | 1.237 | 0.564 |
| **Depression** (no) | - | - | - | - |  | 1.411 | 1.048 | 1.900 | 0.023 |

Notes: Regression analyses conducted by using a newer cut-off of 5.1 mg/dl for the definition of hyperuricemia for females. Models are adjusted on age, marital status, educational level, residence place, region, and social health insurance at baseline (model 1) and other confounding factors (smoking, alcohol drinking, other NCDs, and depression) (model 2). CMD, cardiometabolic disease; RR, Relative risk ratio; CI, confidence interval; Other NCDs, other non-communicable diseases excluding the seven cardiometabolic diseases in this study.

**Table S2**. Association of CMD multimorbidity with all-cause death among participants living the rural and urban area

| **Variable (reference)** |  | **Rural** | |  |  |  | **Urban** | |  |
| --- | --- | --- | --- | --- | --- | --- | --- | --- | --- |
|  | RR | 95% CI | | P value |  | RR | 95% CI | | P value |
| **CMD Multimorbidity** (single disorder) | 1.450 | 1.024 | 2.053 | 0.037 |  | 1.554 | 0.900 | 2.684 | 0.113 |
| **Age** (45-59 years) |  |  |  |  |  |  |  |  |  |
| 55-65 | 1.404 | 0.699 | 2.819 | 0.340 |  | 1.636 | 0.651 | 4.114 | 0.295 |
| 65-75 | 6.626 | 3.512 | 12.500 | <0.001 |  | 4.597 | 1.828 | 11.565 | 0.001 |
| ≥75 | 13.950 | 6.962 | 27.952 | <0.001 |  | 11.511 | 4.122 | 32.142 | <0.001 |
| **Marital status** (married) | 1.344 | 0.902 | 2.001 | 0.146 |  | 1.260 | 0.697 | 2.278 | 0.445 |
| **Education level** (Illiterate) |  |  |  |  |  |  |  |  |  |
| Primary school | 0.733 | 0.398 | 1.349 | 0.318 |  | 1.133 | 0.572 | 2.242 | 0.720 |
| Secondary school | 1.087 | 0.482 | 2.450 | 0.841 |  | 1.347 | 0.628 | 2.889 | 0.444 |
| College and above | 0.675 | 0.092 | 4.982 | 0.700 |  | 0.248 | 0.033 | 1.874 | 0.177 |
| **Region (**east**)** |  |  |  |  |  |  |  |  |  |
| Central | 0.968 | 0.644 | 1.454 | 0.874 |  | 1.273 | 0.694 | 2.337 | 0.436 |
| West | 1.166 | 0.750 | 1.814 | 0.495 |  | 1.618 | 0.828 | 3.165 | 0.159 |
| **Social health insurance** (no) | 0.615 | 0.359 | 1.055 | 0.077 |  | 0.632 | 0.309 | 1.293 | 0.209 |
| **Smoking** (no) | 1.095 | 0.552 | 2.175 | 0.795 |  | 1.639 | 0.730 | 3.682 | 0.231 |
| **Alcohol drinking** (no) | 1.185 | 0.734 | 1.914 | 0.487 |  | 0.351 | 0.085 | 1.446 | 0.147 |
| **Other NCDs** (no) | 0.774 | 0.538 | 1.114 | 0.168 |  | 1.317 | 0.760 | 2.283 | 0.326 |
| **Depression** (no) | 1.293 | 0.903 | 1.852 | 0.161 |  | 1.722 | 1.017 | 2.918 | 0.043 |

Notes: Regression analyses conducted by using a newer cut-off of 5.1 mg/dl for the definition of hyperuricemia for females. Models are adjusted on age, marital status, educational level, region, social health insurance, smoking, alcohol drinking, other NCDs, and depression. CMD, cardiometabolic disease; RR, Relative risk ratio; CI, confidence interval; Other NCDs, other non-communicable diseases excluding the seven cardiometabolic diseases in this study.

**Table S3.** Association of single disorder with all-cause death

| **Variable** |  | RR | 95% CI | | P value |
| --- | --- | --- | --- | --- | --- |
| Heart disease |  | 2.912 | 1.931 | 4.391 | <0.001 |
| Stroke |  | 1.384 | 1.037 | 1.847 | 0.028 |
| Diabetes |  | 1.667 | 1.242 | 2.237 | 0.001 |
| Hypertension |  | 1.019 | 0.800 | 1.298 | 0.880 |
| Hyperlipidaemia |  | 1.004 | 0.782 | 1.289 | 0.976 |
| Hyperuricemia |  | 1.227 | 0.867 | 1.737 | 0.248 |
| Concentric obesity |  | 0.958 | 0.521 | 1.761 | 0.890 |

Notes: Regression analyses are adjusted on age, marital status, educational level, residence place, region, social health insurance, smoking, alcohol drinking, other NCDs, and depression. RR, Relative risk ratio; CI, confidence interval.
